# Host insect species of *Ophiocordyceps* sinensis: a review

**DOI:** 10.3897/zookeys.127.802

**Published:** 2011-09-08

**Authors:** Xiao-Liang Wang, Yi-Jian Yao

**Affiliations:** 1 State Key Laboratory of Mycology, Institute of Microbiology, Chinese Academy of Sciences, P.O. Box 2714, Beijing 100101, P.R. China; 2 Graduate University of the Chinese Academy of Sciences, Beijing 100049, P.R. China

**Keywords:** *Cordyceps*, Fungi, Hepialidae, host insects, *Ophiocordyceps*

## Abstract

*Ophiocordyceps sinensis* (≡ *Cordyceps sinensis*) is one of the most valued medicinal fungi in China, used for its invigorating effects in strengthening the body and restoring energy. The fungus parasitizes larvae of moths and converts them into sclerotia from which the fungus fruiting body grows. Since the late 1950s, considerable effort has been devoted to the study of host insects related to the fungus. In the present paper, the research history of insect species associated with *Ophiocordyceps sinensis* is briefly reviewed and an extensive literature survey is presented. Ninety-one insect names, spanning 13 genera, related to host insects of *Ophiocordyceps sinensis* are investigated. The relationships between the reported insect species and *Ophiocordyceps sinensis* are analyzed. Fifty-seven of these are considered as recognizable potential host species of the fungus distributed throughout the Tibetan Plateau, whilst eight are considered as indeterminate hosts and 26 as non-hosts. Among the names of recognizable potential host insects, three are invalid (*nomen nudum*) and require further study. This work provides basic information for management of the insect resources and for the conservation and sustainable use of *Ophiocordyceps sinensis*.

## Introduction

*Ophiocordyceps sinensis* (Berk.) G.H. Sung, J.M. Sung, Hywel-Jones & Spatafora is an ascomycete fungus, which is also known as the Chinese Caterpillar Fungus or “Dong Chong Xia Cao” (winter worm, summer grass) in Chinese, or “Hia Tsao Tong Tchong” and “Hea Tsaon Tsong Chung” in early English translations (Pegler et al. 1994). The fungus parasitizes larvae of moths belonging to the order Lepidoptera, especially *Hepialus*/*Thitarodes*. The infected larva is converted into a sclerotium covered by the intact exoskeleton of the insect to withstand the winter, which is regarded as “winter worm”. In the late spring or summer of the next year, a clavate stroma of the fungus grows from the sclerotium and emerged from the ground appearing as a herb, which is regarded as “summer grass” (Pegler et al. 1994, Yao 2004). As a valued Chinese herb and tonic, *Ophiocordyceps sinensis* has a long history of use and a high reputation of value both in China and abroad. In Traditional Chinese Medicine (TCM), the fungus is believed to nourish the lungs and kidneys (Wu 1757). It has also been shown in recent studies to have multiple pharmacological effects, including immunomodulating (Wu et al. 2006), hypocholesterolemic (Koh et al. 2003), hypoglycemic (Zhang et al. 2006), anti-tumor (Wu et al. 2005), anti-oxidation (Dong and Yao 2008) and anti-aging (Ji et al. 2009) activities.

The natural product of *Ophiocordyceps sinensis* for medicinal use is actually a combination of the fungus and an insect larva. The fungus parasitizes underground dwelling larvae of moths and converts them into sclerotia, from which the fruiting body of the fungus grows (Pegler et al. 1994, Wang 1995, Yao 2004). *Ophiocordyceps sinensis* is endemic to the Tibetan Plateau, with a distribution covering five provinces in China, i.e., Gansu, Qinghai, Sichuan, Tibet and Yunnan. It may be found in alpine meadow and shrub habitat from an altitude of 3000 m up to the snow-line (Wang 1995, Yao 2004). The natural production of the fungus is limited owing to its strict host-specificity, confined geographic distribution and over exploitation by humans in recent decades. It is therefore currently listed as an endangered species under the second class of state protection (State Forestry Administration and Ministry of Agriculture 1999).

Insect host species of *Ophiocordyceps sinensis* belong to the family Hepialidae (Lepidoptera) (Chu et al. 2004). Since the late 1950s, much effort has been devoted to study the insect species related to the fungus in China. In 1958, researchers from the Institute of Zoology, Chinese Academy of Sciences, began their investigation in some parts of Qinghai and Sichuan provinces. The first report on host insects of *Ophiocordyceps sinensis* in China was on *Hepialus armoricanus* Oberthür (=*Thitarodes armoricanus* Oberthür, Chu 1965) and then followed by studies of biological characteristics of the moth (Chen et al. 1973). The research on Hepialidae diversity and taxonomy grew rapidly in China during the 1980s, leading to a series of publications of new taxa, including four new genera, 71 new species and one subspecies (see Chu and Wang 1985a, b; Liang et al. 1988; Wang 1990; Wu 1992; Li et al. 1993; Fu et al. 1991, 2002; Yang 1993, 1994; Yang et al. 1991a, 1992a, 1995; Liang 1995; Yang and Jiang 1995; Shen and Zhou 1997; Yan 2000; Wang et al. 2001; Chu et al. 2004; Zhang et al. 2007; Tu et al. 2009). A number of attempts have been made to summarize the insect species associated with *Ophiocordyceps sinensis* and various numbers of host species were recorded, e.g., five by Yin (1987); eleven by Yin et al. (1991) without a name list; two, 22 and 23 by Jiang (1989, 1991, 2001); 22 by Chen and Jin (1992); 20 by Long (1992); 37 by Liu et al. (1995); 19 by Li (1996); 38 by Dong and Luo (1996); 31 by Wang et al. (1996) and 37 by Yang (1998). Recently, Chu et al. (2004) published the volume on Hepialidae and Epiplemidae in the Fauna Sinica, in which seven genera and 82 species or subspecies of Hepialidae in China were listed, and 14 species in six genera were believed to be hosts of *Ophiocordyceps sinensis* but the names were not given in that list. However, in two other recent publications (Liu et al. 2005, 2006), 66 and 69 insect names were listed respectively as the hosts of the fungus. However, all these accounts, except Chu et al. (2004), provided only the number or a list of insect names without any relevant information to determine whether they are hosts of the fungus or not. Therefore, the number of insect host species of *Ophiocordyceps sinensis* and the relationship between those insects and the fungus remain unclear. To clarify this situation, an extensive survey of the literature on the host of *Ophiocordyceps sinensis* was carried out to gather all the insect names related to the fungus in the literature and to analyze the relationship between the insect species and the fungus. The results of this work are reported here.

Recently, a global inventory of the suborder Exoporia, comprising Mnesarchaeoidea and Hepialoidea, was presented by Nielsen et al. (2000), in which the systematic position of many taxa was checked and adjusted. Nielsen et al.’s classification system for Hepialidae is adopted in this study.

## Methods

Based upon an exhaustive literature search, a total of 4793 publications related to *Cordyceps*/*Ophiocordyceps* and *Hepialus*/*Thitarodes*, in either English or Chinese, were gathered. Those publications relevant to host insects of *Ophiocordyceps sinensis*, including reports on taxonomy, checklists, fauna, biological characteristics, ecology and geographical distribution were examined for information about these insects. All the insect names associated with *Ophiocordyceps sinensis* were assessed based on the following criteria to determine their relationship with the fungus. Taxa which met both of the following requirements were considered as recognizable potential insect host species of *Ophiocordyceps sinensis*:(1) The distribution areas of the insect overlapped that of *Ophiocordyceps sinensis*, which was determined on the basis of field collections made by this research group during the years 2000−2010, examination of herbarium specimens, and another exhaustive literature analysis carried out in this laboratory (Li et al. in press). (2) The insect was reported from an altitude above 3000 m on the Tibetan Plateau. However, stem-boring insects were excluded as hosts of the fungus, even if they were hepialid and distributed above 3000 m within the distribution areas of *Ophiocordyceps sinensis*, because the fungus infects only subterranean root-boring insects. Species of root-borers lacking altitude information were considered as indeterminate hosts of *Ophiocordyceps sinensis* requiring further confirmation, despite the overlap of distribution areas with *Ophiocordyceps sinensis*. Species falling in both of the following circumstances were deemed not to be host insects of *Ophiocordyceps sinensis*: the distribution of the insect was outside that of *Ophiocordyceps sinensis* and below an altitude of 3000 m.

## Results

A total of 91 names in 13 genera of Hepialidae were found in the literature search. They are listed in alphabetical order in Table 1, together with geographic distribution, altitude, main references and the relationship with *Ophiocordyceps sinensis* as determined by this study. Insect names used in the references, if different from that in Nielsen et al. (2000), are also given. There are 67 names in the references being combined in different genera by Nielsen et al. (2000) and a total of 71 species were originally described from China. Twenty four species described in the literature were not included in Nielsen et al. (2000).

Fifty-seven species are considered here as recognizable potential host insects of *Ophiocordyceps sinensis*, whilst eight as indeterminate hosts and 26 as non-hosts. The recorded altitude ranges of the recognized potential host insects were found to vary from 2800 to 5100 m. The distribution areas of these species covered 26 provinces in China and more than 12 other countries. Three of the recognizable potential host names are invalid (*nomen nudum*).

**Table:** **Table 1.** Potential insect hosts of *Ophiocordyceps sinensis*.

Insect name	Geographic distribution	Altitude (m)	Main references	Status of host insect†	Different name in the main references
*Bipectilus yunnanensis* Chu & Wang, 1985	Yunnan Province: Lijiang County‡	3200	Chu and Wang 1985a, Nielsen 1988	P	
*Bipectilus zhejiangensis* Wang, 2001§	Zhejiang Province: Anji County‡; Fujian Province	—	Wang et al. 2001, Huang 2006, Wu 2007	N	
*Endoclita anhuiensis* (Chu & Wang, 1985)|	Anhui Province: Yuexi County‡	—	Chu and Wang 1985b, Chu et al. 2004	N	*Phassus anhuiensis* Chu & Wang, 1985
*Endoclita davidi* (Poujade, 1886)	Sichuan Province: Baoxing and Danba Counties; Fujian and Guangxi Provinces	3600	Chu and Wang 1985b, Yang 1998, Chu et al. 2004	P	*Hepialus davidi* Poujade, 1886;*Phassus giganodus* Chu & Wang, 1985
*Endoclita excrescens* (Butler, 1877)|	Sichuan Province: Yingjing County; Anhui, Hebei, Heilongjiang, He’nan, Jilin, Liaoning, Shandong and Shanxi Provinces; Inner Mongolia Autonomous Region; Japan	—	Chu et al. 2004	N	*Phassus excrescens* (Butler, 1877) ;*Phassus camphorae* Sasaki, 1908
*Endoclita fijianodus* (Chu & Wang, 1985)|	Fujian Province‡	—	Chu and Wang 1985b, Chu et al. 2004	N	*Phassus fujianodus* Chu & Wang, 1985
*Endoclita jingdongensis* (Chu & Wang, 1985)|	Yunnan Province: Jingdong County‡, Xishuangbanna Prefecture‡	—	Chu and Wang 1985b, Chu et al. 2004	N	*Phassus jingdongensis* Chu & Wang, 1985
*Endoclita nodus* (Chu & Wang, 1985)|	Anhui Province: Yuexi County‡; Guangxi, Guizhou, Hainan, Hu’nan, Jiangxi and Zhejiang Provinces	—	Chu and Wang 1985b, Chu et al. 2004	N	*Phassus nodus* Chu & Wang, 1985
*Endoclita signifer* (Walker, 1856)|	Hu’nan Province	—	Chu and Wang 1985b, Chu et al. 2004	N	*Phassus hunanensis* Chu & Wang, 1985
*Endoclita sinensis* (Moore, 1877)|	Fujian, Guangdong, Guangxi, Hainan, Hebei, He’nan, Hubei, Hu’nan, Jiangxi, Shandong, Shanxi, Sichuan, Yunnan and Zhejiang Provinces; Shanghai Municipality; D.P.R. Korea; India; Japan; Sri Lanka	—	Chu et al. 2004	N	*Phassus sinensis* Moore, 1877;*Phassus herzi* Fixsen, 1887
*Endoclita xizangensis* (Chu & Wang, 1985)|	Tibet Autonomous Region: Nyalam County‡	—	Chu and Wang 1985b, Wang et al. 1996, Chu et al. 2004	N	*Phassus xizangensis* Chu & Wang, 1985
*Endoclita yunnanensis* (Chu & Wang, 1985)|	Yunnan Province: Jinghong Municipality‡; Guangdong and Hainan Provinces	—	Chu and Wang 1985b, Chu et al. 2004	N	*Phassus yunnanensis* Chu & Wang, 1985
*Gazoryctra ganna* (Hübner, [1808])	Qinghai Province: Zadoi County; Heilongjiang Province; Inner Mongolia Autonomous Region; Northern Europe; Russia	3900¶	Chu and Wang 1985a, Wang et al. 1996, Yang 1998, Chu et al. 2004, Karsholt and Nieukerken 2010	P	*Hepialus ganna* (Hübner, [1808])
*Gazoryctra macilentus* (Eversmann, 1851)	Hebei and Heilongjiang Provinces; Inner Mongolia Autonomous Region; Eastern Siberia; Mongolia	340–1300	Wang et al. 1996, Yang 1998, Chu et al. 2004	N	*Hepialus macilentus* Eversmann, 1851
*Hepialiscus jiangbeiensis* Chu & Wang, 2004§	Chongqing Municipality‡	—	Chu et al. 2004	N	
*Hepialiscus ledongensis* Chu & Wang, 2004§	Hainan Province: Ledong County‡	—	Chu et al. 2004	N	
*Hepialiscus nepalensis* (Walker, 1856)	Tibet Autonomous Region: Nyalam County; India; Nepal; Sikkim	—	Chu and Wang 1985a, Wang et al. 1996, Chu et al. 2004	I	*Hepialiscus flavus* Chu & Wang, 1985
*Hepialus bibelteus* Shen & Zhou, 1997§	Yunnan Province: Deqên County‡	4500	Shen and Zhou 1997, Chu et al. 2004	P	
*Hepialus biruensis* Fu, 2002§	Tibet Autonomous Region: Biru County‡	4400–4700	Fu et al. 2002, Chu et al. 2004	P	
*Hepialus dinggyeensis* Chu & Wang, 2004§	Tibet Autonomous Region: Dinggyê County‡	—	Chu et al. 2004	I	
*Hepialus gangcaensis* Chu & Wang, 2004§	Qinghai Province: Gangca County‡	3195¶	Chu et al. 2004	P	
*Hepialus guidera* Yan, 2001§	Qinghai Province: Guide County	3400–3600	Yan 2001a, Li et al. 2002, Li and Li 2004	P, IN	*Hepialus guidera* Yan, 2001
*Hepialus hainanensis* Chu & Wang, 2004§	Hainan Province: Ledong County‡	—	Chu et al. 2004	N	
*Hepialus humuli* (Linnaeus, 1758)	Heilongjiang Province; Europe and Siberia	—	Chu et al. 2004, Karsholt and Nieukerken 2010	N	
*Hepialus lagii* Yan, 2001§	Qinghai Province: Guide County	3400–3600	Yan 2001b; Yan 2001c, Li et al. 2002, Li and Li 2004, Zhang et al. 2009	P, IN	
*Hepialus latitegumenus* Shen & Zhou, 1997§	Yunnan Province: Deqên County‡	4500	Shen and Zhou 1997, Chu et al. 2004	P	
*Hepialus maquensis* Chu & Wang, 2004§	Gansu Province: Maqu County‡	3300¶	Chu et al. 2004	P	
*Hepialus namensis* Chu & Wang, 2004§	Tibet Autonomous Region: Damxung County‡	4200¶	Chu et al. 2004	P	
*Hepialus namlinensis* Chu & Wang, 2004§	Tibet Autonomous Region: Namling County‡	3704¶	Chu et al. 2004	P	
*Hepialus pui* Zhang, Gu & Liu, 2007§	Tibet Autonomous Region: Nyingchi County‡	4100–5000	Zhang et al. 2007	P	
*Hepialus xiaojinensis* Tu, Ma & Zhang 2009§	Sichuan Province: Xiaojin‡ and Jinchuan County‡	3500–4800	Tu et al. 2009	P	
*Hepialus xingazeensis* Chu & Wang, 2004§	Tibet Autonomous Region: Xigazê Prefecture‡	—	Chu et al. 2004	I	
*Hepialus yadongensis* Chu & Wang, 2004§	Tibet Autonomous Region: Yadong County‡	—	Chu et al. 2004	I	
*Hepialus yongshengensis* Chu & Wang, 2004§	Yunnan Province: Yongsheng County‡	—	Chu et al. 2004	I	
*Hepialus zadoiensis* Chu & Wang, 2004§	Qinghai Province: Zadoi County‡	3900¶	Chu et al. 2004	P	
*Magnificus jiuzhiensis* Yan, 2000§	Qinghai Province: Jigzhi County‡	3800–3900	Yan 2000	P	
*Magnificus zhiduoensis* Yan, 2000§	Qinghai Province: Zhidoi County‡	4400–4600	Yan 2000	P	
*Napialus chenzhouensis* Chu & Wang, 2004§	Hu’nan Province: Chenzhou City‡; Shanghai Municipality	—	Chu et al. 2004, Chen and Wang 2006	N	
*Napialus chongqingensis* Wu, 1992	Chongqing Municipality‡	—	Wu 1992, Chu et al. 2004	N	
*Napialus hunanensis* Chu & Wang, 1985	Hu’nan Provinces: Changsha City‡; Guangdong, Guangxi, Hainan, and Jiangxi Provinces	—	Chu and Wang 1985a, Wang et al. 1996, Chu et al. 2004	N	
*Napialus jiangxiensis* Chu & Wang, 2004§	Jiangxi Province: Taihe County‡	—	Chu et al. 2004	N	
*Palpifer sexnotatus* (Moore, 1879)|	Sichuan and Taiwan Provinces; Kashmir; India; Sri Lanka; Japan	—	Chu et al. 2004	N	
*Parahepialiscus borneensis* (Pfitzner in Pfitzner & Gaede, 1933)	Hu’nan Province; Malaysia	—	Chu et al. 2004	N	*Hepialiscus borneensis* Pfitzner, 1933
*Pharmacis carna* ([Denis & Schiffermüller], 1775)	Sichuan Province: Luhuo County; Europe	3050¶	Chu et al. 2004, Karsholt and Nieukerken 2010	P	*Hepialus carna* ([Denis & Schiffermüller], 1775)
*Pharmacis fusconebulosa* (De Geer, 1778)	Sichuan Province: Kangding District; Europe; Russia	3500¶	Chu et al. 2004, Yu 2004, Karsholt and Nieukerken 2010	P	*Hepialus fusconebulosa* (De Geer, 1778);*Hepialus gallicus* Lederer, 1852
*Pharmacis pyrenaicus* (Donzel, 1838)	Sichuan Province: Dêgê County; Southwest Europe	3880¶	Chu et al. 2004, Karsholt and Nieukerken 2010	P	*Hepialus alticola* Oberthür, 1881
*Sthenopis regius* (Staudinger, 1896)|	—	—	Yin 1987	N	*Phassus regius* (Staudinger, 1896)
*Sthenopis roseus* (Oberthür, 1911)|	Hubei Province	—	Chu and Wang 1985b, Chu et al. 2004	N	*Phassus miniatus* Chu & Wang, 1985
*Thitarodes albipictus* (Yang, 1993)	Yunnan Province: Deqên County‡	4500–4800	Wang et al. 1996, Yang 1993	P	*Hepialus albipictus* Yang, 1993
*Thitarodes altaicola* (Wang, 1990)	Xinjiang Uygur Autonomous Region‡	1300–l800	Wang 1990, Yang 1998, Chu et al. 2004	N	*Hepialus altaicola* Wang, 1990
*Thitarodes anomopterus* (Yang, 1994)	Yunnan Province: Jianchuan‡ and Lijiang Counties‡	2800–3100	Yang 1994, Yang 1998	P	*Hepialus anomopterus* Yang, 1994
*Thitarodes armoricanus* (Oberthür, 1909)	Gansu, Qinghai, Sichuan and Yunnan Provinces; Tibet Autonomous Region; Xinjiang Uygur Autonomous Region	3600–5000	Chu 1965, Chen et al. 1973, Yang et al. 1987, Chu et al. 2004	P	*Hepialus armoricanus* Oberthür, 1909
*Thitarodes baimaensis* (Liang in Liang et al. 1988)	Yunnan Province: Deqên County‡	4500–4900	Liang et al. 1988, Yang 1998	P	*Hepialus baimaensis* Liang, 1988
*Thitarodes baqingensis* (Yang & Jiang, 1995)	Tibet Autonomous Region: Baqên County‡	4600–4800	Yang and Jiang 1995	P	*Hepialus baqingensis* Yang & Jiang, 1995
*Thitarodes callinivalis* (Liang, 1995)	Tibet Autonomous Region; Yunnan Province : Deqên County‡	4300–4600	Liang 1995, Yang 1998	P	*Hepialus callinivalis* Liang, 1995
*Thitarodes cingulatus* (Yang & Zhang in Yang et al. 1995)	Gansu Province: Wenxian County‡	3200–3800	Yang et al. 1995, Yang 1998	P	*Hepialus cingulatus* Yang & Zhang, 1995
*Thitarodes damxungensis* (Yang in Yang and Jiang 1995)	Tibet Autonomous Region: Damxung County‡	4500–4680	Yang and Jiang 1995	P	*Hepialus damxungensis* Yang, 1995
*Thitarodes deqinensis* (Liang in Liang et al. 1988)	Yunnan Province: Deqên County	4200–4700	Liang et al. 1988, Yang et al. 1992b	P	*Hepialus deqinensis* Liang, 1988
*Thitarodes dongyuensis* (Liang in Yang et al. 1992)	Tibet Autonomous Region: Markam County; Yunnan Province: Deqên County	4000–4700	Yang et al. 1992b, Yang et al. 1996, Hu and Zha 2010	P, IN	*Hepialus dongyuensis* Liang in Yang et al. 1992
*Thitarodes ferrugineus* (Li, Yang & Shen, 1993)	Yunnan Province: Deqên County‡	4200–4700	Yang et al. 1992b, Li et al. 1993, Chu et al. 2004	P	*Hepialus ferrugineus* Li, Yang & Shen, 1993
*Thitarodes gonggaensis* (Fu & Huang in Fu et al., 1991)	Sichuan Province: Kangding County‡	3800–4400	Fu et al. 1991, Yang 1998, Chu et al. 2004	P	*Hepialus gonggaensis* Fu & Huang, 1991
*Thitarodes jialangensis* (Yang, 1994)	Tibet Autonomous Region: Zogang County‡	4000–4600	Yang 1994, Chu et al. 2004	P	*Hepialus jialangensis* Yang, 1994
*Thitarodes jianchuanensis* (Yang, 1994)	Yunnan Province: Jianchuan County‡	2900–3500	Yang 1994, Yang 1998	P	*Hepialus jianchuanensis* Yang, 1994
*Thitarodes jinshaensis* (Yang, 1993)	Yunnan Province: Deqên County‡	4600	Yang 1993, Chu et al. 2004	P	*Hepialus jinshaensis* Yang, 1993
*Thitarodes kangdingensis* (Chu & Wang, 1985)	Sichuan Province: Kangding County‡	3600–4500	Chu and Wang 1985a, Yang et al. 1991b, Chu et al. 2004	P	*Hepialus kangdingensis* Chu & Wang, 1985
*Thitarodes kangdingroides* (Chu & Wang, 1985)	Sichuan Province: Kangding County‡	4200	Chu and Wang 1985a, Yang 1998, Chu et al. 2004	P	*Hepialus kangdingroides* Chu & Wang, 1985
*Thitarodes lijiangensis* (Chu & Wang, 1985)	Yunnan Province: Lijiang County‡	3500–4400	Chu and Wang 1985a, Yang 1998, Chu et al. 2004	P	*Hepialus lijiangensis* Chu & Wang, 1985
*Thitarodes litangensis* (Liang, 1995)	Sichuan Province: Litang‡ and Batang County; Tibet Autonomous Region	4300–4700	Liang 1995, Yang 1996, Yang 1998	P	*Hepialus litangensis* Liang, 1995
*Thitarodes luquensis* (Yang & Yang in Yang et al. 1995)	Gansu Province: Luqu County‡	4276–4300	Yang et al. 1995, Yang 1998	P	*Hepialus luquensis* Yang & Yang, 1995
*Thitarodes markamensis* (Yang, Li & Shen, 1992)	Tibet Autonomous Region: Markam County‡; Yunnan Porvince: Deqên County	4500–4900	Yang et al. 1992a, b, Yang 1998	P	*Hepialus markamensis* Yang, Li & Shen, 1992
*Thitarodes meiliensis* (Liang in Liang et al. 1988)	Yunnan Province: Deqên County‡	3650–4700	Liang et al. 1988, Wang et al. 1996, Yang 1998	P	*Hepialus meiliensis* Liang, 1988
*Thitarodes menyuanicus* (Chu & Wang, 1985)	Gansu Province: Jishishan County; Qinghai Province: Hualong, Menyuan‡ and Tongren Counties	—	Chu and Wang 1985a, Ma et al. 1995, Yang 1998	I	*Hepialus menyuanicus* Chu & Wang, 1985
*Thitarodes nebulosus* (Alpheraky, 1889)	Qinghai Province: Yushu Prefecture; Tibet Autonomous Region: Amdo‡ and Damxung Counties‡, Nagqu Prefecture	4500	Yin 1987, Yang 1998, Chu et al. 2004	P	*Hepialus nebulosus* Alphéraky, 1889
*Thitarodes oblifurcus* (Chu & Wang, 1985)	Qinghai Province: Yushu Prefecture‡; Sichuan Province: Kangding County	4000–4500	Chu and Wang 1985a, Gao et al. 1992, Yang 1998	P	*Hepialus oblifurcus* Chu & Wang, 1985
*Thitarodes pratensis* (Yang, Li & Shen, 1992)	Yunnan Province: Deqên County‡	4350	Yang et al. 1992a	P	*Hepialus pratensis* Yang, Li & Shen, 1992
*Thitarodes renzhiensis* (Yang in Yang et al. 1991)	Yunnan Province: Deqên County‡	3880–5100	Yang et al. 1991a, Wang et al. 1996, Yang 1998	P	*Hepialus renzhiensis* Yang, 1991
*Thitarodes sichuanus* (Chu & Wang, 1985)	Sichuan Province‡: Aba Prefecture, Emei and Kangding Counties; Chongqing Municipality	3600–3800	Chu and Wang 1985a, Wang et al. 1996, Yang 1998, Chu et al. 2004	P	*Hepialus sichuanus* Chu & Wang, 1985
*Thitarodes varians* (Staudinger, 1896)	Sichuan Province: Batang County; Tibet Autonomous Region: Qamdo County	4500	Yin 1987, Yang 1998	P	*Hepialus varians* Staudinger, 1896
*Thitarodes xizangensis* (Chu & Wang, 1985)	Tibet Autonomous Region: Nyalam County‡	2200	Chu and Wang 1985a, Wang et al. 1996	N	*Forkalus xizangensis* Chu & Wang, 1985
*Thitarodes xunhuaensis* (Yang & Yang in Yang et al. 1995)	Qinghai Province: Xunhua County‡	3800	Yang et al. 1995, Yang 1998	P	*Hepialus xunhuaensis* Yang & Yang, 1995
*Thitarodes yeriensis* (Liang, 1995)	Yunnan Province: Deqên County‡	4500–4700	Liang 1995, Yang 1998	P	*Hepialus yeriensis* Liang, 1995
*Thitarodes yulongensis* (Liang, 1988)	Yunnan Province: Lijiang County‡	4150–4500	Liang et al. 1988, Wang et al. 1996, Yang 1998	P	*Hepialus yulongensis* Liang, 1988
*Thitarodes yunlongensis* (Chu & Wang, 1985)	Yunnan Province: Yunlong‡ and Dali Counties; Hainan Province	3600–4200	Chu and Wang 1985a, Wang et al. 1996, Yang 1998, Chu et al. 2004	P	*Hepialus yunlongensis* Chu & Wang, 1985
*Thitarodes yunnanensis* (Yang, Li & Shen, 1992)	Yunnan Province: Jianchuan‡, Lanping‡, Lijiang‡ and Weixi Counties	3600–4100	Yang et al. 1992a, b, Wang et al. 1996, Yang 1998	P	*Hepialus yunnanensis* Yang, Li & Shen, 1992
*Thitarodes yushuensis* (Chu & Wang, 1985)	Qinghai Province: Yushu Prefecture‡, Batang, Chindu and Zadoi Counties; Gansu Province	4500–4900	Chu and Wang 1985a, Yang et al. 1991b, Yang 1998, Ma et al. 1995	P	*Hepialus yushuensis* Chu & Wang, 1985
*Thitarodes zaliensis* (Yang, 1994)	Tibet Autonomous Region: Markam County‡	4600–4900	Yang 1994, Yang 1998	P	*Hepialus zaliensis* Yang, 1994
*Thitarodes zhangmoensis* (Chu & Wang, 1985)	Tibet Autonomous Region: Nyalam County‡	2200	Chu and Wang 1985a, Wang et al. 1996	N	*Hepialus zhangmoensis* Chu & Wang, 1985
*Thitarodes zhayuensis* (Chu & Wang, 1985)	Tibet Autonomous Region: Zayü‡ and Markam Counties; Yunnan Province: Deqên and Gongshan County	4200–4400	Chu and Wang 1985a, Yang et al. 1987, Yang 1998	P	*Hepialus zhayuensis* Chu & Wang, 1985
*Thitarodes zhongzhiensis* (Liang, 1995)	Yunnan Province: Deqên County‡	4000–4600	Liang 1995, Wang et al. 1996	P	*Hepialus zhongzhiensis* Liang, 1995
*Triodia nubifer* (Lederer, 1853)	Sichuan Province: Kangding Prefecture; Central Asia	—	Chu et al. 2004	I	*Hepialus nubifer* Lederer, 1853
*Triodia sylvina* (Linnaeus, 1761)	Sichuan Province: Kangding County; Central Asia; Central Europe and Northern Europe	—	Chu and Wang 1985a, Chu et al. 2004, Karsholt and Nieukerken 2010	I	*Hepialiscus sylvinus* (Linnaeus, 1761)

† The status of host insect of *Ophiocordyceps sinensis* determined in this study: I = indeterminate host, N = non-host, IN = invalid name, P = potential host;

‡ Type-locality;

§ Names not included in Nielsen et al. (2000);

| Stem-borers;

¶ The lowest altitude of the reported locality in China.

## Discussion

Through an extensive literature survey, all the Hepialidae species reported from China were listed and analyzed using detailed information on their geographic distribution, altitude and nomenclature. The relationships between the insect species and *Ophiocordyceps sinensis* were clarified based on available information. The data provided here serve as a foundation for further investigations on the conservation biology of this endangered fungal species and its insect hosts.

Species in different genera of Chinese hepialids can be divided into two categories according to the feeding strategy of the larvae (Chu and Wang 1985a, b; Chu et al. 2004): stem-borers (12 taxa) and root-borers (79 taxa). The stromata of *Ophiocordyceps sinensis* are produced directly on the dead larvae of hepialids which were tunneling under the ground (Wang 1995, Yao 2004, Sung et al. 2007), and the host larvae of the fungus feed on plant roots underground (Chen et al. 1973, Shen et al. 1983, Wang 1995, Yao 2004). Therefore, the stem-borers,including nine in *Endoclita*, one in *Palpifer* andtwo in *Sthenopis* (Table 1), apparently can not be hosts of *Ophiocordyceps sinensis*. The remaining 79 taxa found in this survey were categorized as potential hosts, indeterminate hosts, or non-hosts assessed based on the criteria described in the methods. Fourteen of the 79 root-borers were ruled out as hosts of *Ophiocordyceps sinensis*, including 1 *Bipectilus*, 1 *Gazoryctra*, 2 *Hepialiscus*, 2 *Hepialus*, 4 *Napialus*, 1 *Parahepialiscus* and 3 *Thitarodes* species (Table 1), because they have not been reported from the distribution area of *Ophiocordyceps sinensis* and were found below the elevation of 3000 m, either far away from the Tibetan Plateau (12 species), e.g., *Bipectilus zhejiangensis* from Zhejiang Province, *Hepialus hainanensis* from Hainan Province, etc., or on the Plateau (two species), e.g., *Thitarodes xizangensis* and *Thitarodes zhangmoensis*, which were found in Zhangmu Town in Tibet Autonomous Region, where the altitude range is from 1700 to 2400 m (People’s Government of Tibet Autonomous Region, 2011) and no evidence for the occurrence of *Ophiocordyceps sinensis* has been found (Li et al. in press).

Eight species, including 1 *Hepialiscus*, 4 *Hepialus*, 1 *Thitarodes* and 2 *Triodia* species (Table 1), are considered as indeterminate hosts of *Ophiocordyceps sinensis*. While the distribution ranges of these species are within that of *Ophiocordyceps sinensis*, they lack an altitude record and require further confirmation before being considered as potential hosts of *Ophiocordyceps sinensis*, e.g., *Hepialus yadongensis*, *Triodia sylvina*, etc.

Fifty-seven taxa are recognized as potential hosts of *Ophiocordyceps sinensis*, including 1 *Bipectilus*, 1 *Endoclita*, 1 *Gazoryctra*, 12 *Hepialus*, 2 *Magnificus*, 3 *Pharmacis* and 37 *Thitarodes* species (Table 1). The distribution ranges of these insects overlap that of *Ophiocordyceps sinensis*. Altitude information for these insects was reported in three ways in the literature: (1) The altitude range of the insect was reported unambiguously above 3000 m, e.g., *Thitarodes baimaensis*, *Thitarodes meiliensis*, etc. (37 species). Among these, the lowest altitude of 3200 m was reported for *Thitarodes cingulatus* (Yang 1998). (2) The altitude range of the species was not specified, but the types were collected at an elevation above 3000 m, e.g., *Thitarodes baqingensis*, *Magnificus jiuzhiensis*, etc. (11 species). The lowest altitude of the type locality is at 3200 m for *Bipectilus yunnanensis* (Chu and Wang 1985a). (3) There is no data reported on the altitude range for the species or the type specimen, but the altitude of the recorded localities of the moth were above 3000 m, e.g., *Hepialus gangcaensis*, *Pharmacis carna*, etc. (nine species). The lowest altitude for the locality of this group is 3050 m for *Pharmacis carna* (Chu et al. 2004) in Luhuo County, Sichuan Province, where the occurrence *Ophiocordyceps sinensis* was confirmed (Li et al. in press).

Three names of the recognizable potential host insects are invalid (*nomen nudum*) because no full description of the species was published in the literature, although the names appeared several times in various publications (Table 1). Among them, *Thitarodes dongyuensis* was described by Yang (1992) as ‘*Hepialus dongyuensis*’ and deemed as a *nomen nudum* in Nielsen et al. (2000), while *Hepialus guidera* and *Hepialus lagii* were described by Yan (2001a, b) and recognized as *nomen nudum* in the present study. Further study is required to describe these species in full.

Species of *Hepialus* and *Phassus* described from China after 1984 have been transferred to *Thitarodes* and *Endoclita* respectively by Nielsen et al. (2000). Most of these species were described on the male genitalia and occasionally venation of one or very few individuals but not all morphological characteristics of the adult (Nielsen et al. 2000). However, the structure of the valve on male genitalia was still employed recently as the sole basis for classification in the revision of Chinese *Hepialus* by Zou et al. (2010). Further, disparate and incongruent regional taxonomies were regarded as developing rapidly for the Chinese Hepialidae (Nielsen et al. 2000), but the situation has not been changed much. As seen in this study, 24 names listed in Table 1 were not included in Nielsen et al. (2000). Two of them were described pre-2000 and apparently missed by Nielsen and his colleagues, while the remaining 22 were newly described after the year 2000 (Table 1). It seems that further study, especially robust phylogenetic hypotheses from molecular data, of these taxa is required to clarify their taxonomic status and generic placement.

Natural production of *Ophiocordyceps sinensis* has been declining significantly over the last few decades while the market demands on the fungus have increased sharply in recent years. Clarification of the host insects of *Ophiocordyceps sinensis* will provide basic information for management of the insect resources and for the conservation and sustainable use of the fungus. This work has gathered the available information on the host insects of *Ophiocordyceps sinensis* and will lay a foundation for further studies of the relationship between the fungus and its hosts, especially their co-evolution (an ongoing research project based on DNA sequence analyses in this laboratory), and also for the cultivation of this valuable fungus for massive production.
